# A Case of Fetal Parvovirus B19 Myocarditis That Caused Terminal Heart Failure

**DOI:** 10.1155/2014/463571

**Published:** 2014-09-25

**Authors:** Atsuko Hichijo, Mikio Morine

**Affiliations:** ^1^Department of Obstetrics and Gynecology, Institute of Health Biosciences, The University of Tokushima Graduate School, Tokushima 770-8503, Japan; ^2^Department of Maternal Fetal Medicine, Center for Maternal, Fetal and Neonatal Medicine, Shikoku Medical Center for Children and Adults, 2-1-1 Zentsuji, Kagawa 765-8507, Japan

## Abstract

Parvovirus B19 is a well-established cause of fetal anemia and nonimmune fetal hydrops in pregnancy. Fetal parvovirus infection can cause severe destruction of erythroid progenitor cells, resulting in fetal anemia, hydrops, and intrauterine death. However, viral myocarditis with subsequent heart failure is another possible mechanism for hydrops formation as viral infection of fetal myocardial cells has been reported in postmortem examinations. We herein report a case of fetal cardiomegaly and massive pericardial effusion secondary to myocarditis as a result of parvovirus B19 infection. The case developed hydrops as consequence of severe anemia and experienced terminal heart failure, which led to the fetus dying an intrauterine death at 22 weeks of gestation. This case demonstrates that there may be an association between myocarditis caused by intrauterine parvovirus B19 infection and a poor outcome. The presence of viral myocarditis may be the determining prognostic factor in that situation.

## 1. Introduction

Human parvoviruses have long been regarded as agents of reproductive failure and parvovirus B19 was recognized as a cause of fetal loss in humans in the 1980s [1]. Human parvovirus B19, a small single-stranded DNA genome approximately 5600 nucleotides in length, causes a wide spectrum of clinical complications, ranging from mild and self-limiting erythema in immunocompetent children to lethal pancytopenia in immunocompromised patients [2]. During pregnancy, parvovirus B19 infection can be asymptomatic or cause a variety of signs of fetal damage, such as severe anemia, nonimmune fetal hydrops, and death. Parvovirus B19 accounts for up to 8–10% of cases of nonimmune fetal hydrops in anatomically normal fetuses [3]. Potential mechanisms underlying the development of fetal hydrops are anemia, myocarditis, and hypoalbuminemia as a result of hepatitis. Infection of fetuses is especially damaging between 10 and 20 weeks of gestation. During this time, the major development of the erythroid precursors takes place and parvovirus B19 infections lead to an arrest of maturation of these cells at the late normoblast stage and extremely low hemoglobin levels have been reported in the affected cases. A fetal parvovirus infection can also cause severe destruction of erythroid progenitor cells, resulting in fetal anemia, hydrops, and intrauterine death. However, it has been unknown whether parvovirus B19 plays a pathogenic role in this condition. Viral myocarditis with subsequent heart failure is another possible mechanism for hydrops formation, as viral infection of fetal myocardial cells has been reported in postmortem examinations.

We herein report a case of fetal hydrops associated with terminal heart failure caused by myocarditis due to an intrauterine parvovirus B19 infection. The aim of the present report is to identify the prognostic factors and suggest effective management for future cases.

## 2. Case Report

**Figure 1:**
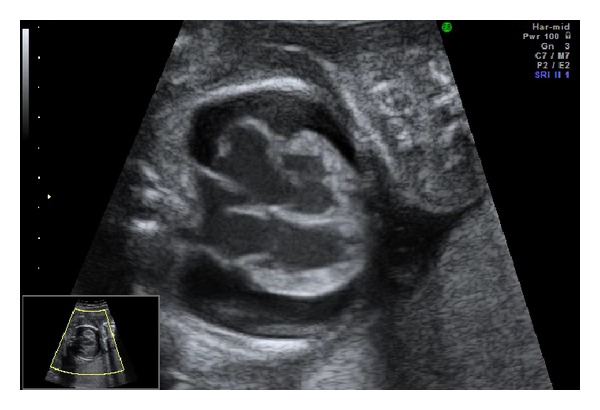
Fetal echocardiography at 21 weeks of gestation. A 2D image of the four-chamber view showed cardiomegaly, dilated ventricles, and atria with thickened and echo-dense walls, as well as massive pericardial effusion.

A 20-year-old primigravida female was referred to our tertiary center at 21 weeks of gestation for the management of fetal hydrops which was characterized by generalized edema, massive pericardial effusion, mild cardiomegaly, and ventricular hypertrophy. Doppler studies showed a high peak systolic velocity in the middle cerebral artery (MCA) of 61.41 cm/s (2.28 MoM) suggestive of fetal anemia. Doppler investigations showed reverse flows in the MCA and ductus venosus (DV) with umbilical venous pulsation. Fetal echocardiography showed cardiomegaly (cardiothoracic area ratio; CTAR 45%) resulting in severe regurgitation of all valves and impaired ventricular function without structural cardiac defects. The endocardium was echo-dense, suggesting the presence of fibroelastosis (Figure 1).

The maternal blood was analyzed for toxoplasma, cytomegalovirus, herpes simplex virus, coxsackie virus, and parvovirus B19 and showed evidence of parvovirus B19 seroconversion. All other infection screening tests were negative for recent infections. The mother did not recall any symptoms of viral infection at the beginning of her pregnancy. The maternal blood groupwas A positive and the maternal red cell antibody screening was negative.

The options for pericardial effusion aspiration and umbilical blood sampling for anemia were discussed. In view of the fetal circulatory disorder and lung decompression, it was decided to perform pericardial centesis and draining of the pericardial effusion. The procedure was performed at 22 weeks of gestation without any complications. However, the fetus died one day later. Fetal pericardial fluid and ascites, as well as the amniotic fluid, tested positive for parvovirus B19 DNA and revealed a normal female karyotype 46, XX.

**Figure 2:**
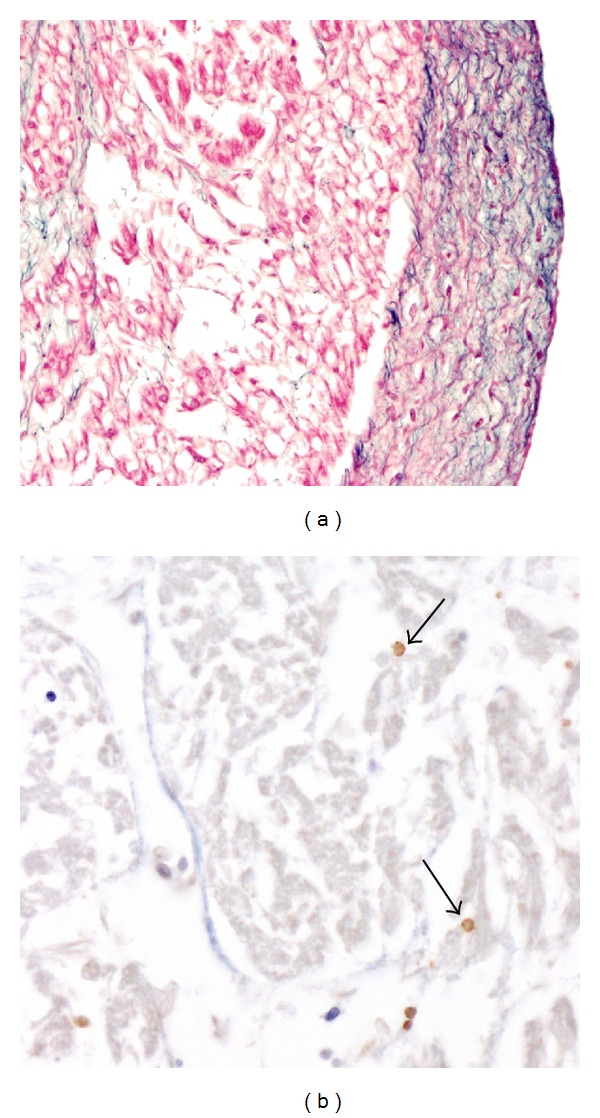
Histopathology obtained during the infant autopsy. (a) Cardiac hematoxylin and eosin staining showed dilatation and massive endocardial fibroelastosis appearing as a thick white scale covering the inner surface of the left ventricle cavity. (b) The immunohistochemical analysis showed extensive inflammatory cell (arrow) infiltrates in the myocardium.

A postmortem examination revealed a hydropic stillborn fetus, weighing 486 g without any gross anomalies. Autopsy of the heart revealed severe hypertrophy and dilatation of the right and left ventricles. The dilated wall of the ventricle was almost circumferentially covered by a white scale of fibrous tissue and extensive inflammatory cell infiltrates were noted (Figure 2). The presence of endocardial fibroelastosis and myocarditis were confirmed by histology. The myocardial and hepatic tissues were investigated for parvovirus B19 RNA using polymerase chain reaction (PCR).

## 3. Discussion

Parvovirus B19 is a member of the parvoviruses family and is the only strain that is pathogenic to humans. The virus is not fatal in adults but is recognized as a potential risk factor for an adverse pregnancy outcome, including fetal hydrops and fetal death, if a nonimmune mother becomes infected during pregnancy. The rate of fetal infection is 33% [4] and the risk of fetal death after maternal exposure is 9% to 26% [1, 5]. The clinical outcome of fetal infection depends on the gestational age, with the greatest risk of embryopathy following maternal infection in the first 20 weeks [4, 5]. Fetal parvovirus B19 infection usually presents as fetal anemia, skin edema, ascites, pleural effusions, and cardiomegaly.

Molecular techniques investigating the amniotic fluid and ascites, pleural effusions, and pericardial effusion of the fetus demonstrated an intrauterine parvovirus B19 infection in the present patient. Parvovirus B19 is highly tropic to human bone marrow and replicates only in erythroid progenitor cells [6]. The basis of this erythroid tropism is the tissue distribution of the B19 cellular receptor, globoside (blood group P antigen) [7, 8]. The P antigen is also found on fetal cardiac myocytes consistent with evidence [9], that a fetus infected with parvovirus B19 can develop myocarditis [10, 11].* In situ* hybridization detected viral DNA sequences in the nuclei of infected myocarditis, which may occur in the absence of fetal anemia. Immunochemical and histological examination of fetuses with parvovirus B19 infection have demonstrated viral involvement of the myocardial cells, thus suggesting that the virus exists within the cardiac tissue itself and that intrauterine myocarditis from direct infection of the myocardium contributes to the development of the nonimmune fetal hydrops [9, 11–16].

We herein reported a case of fetal hydrops associated with terminal heart failure caused by myocarditis due to an intrauterine parvovirus B19 infection and provided evidence of viral DNA in cardiac tissue and the pericardial effusion. Sonographically, fetal myocarditis presents as a thickened, hyperechogenic, and poorly contractile myocardium that has a poor ejection fraction, with or without associated tricuspid incompetence. Therefore, we speculated that in the present fetus with myocarditis and pericarditis, endocardial fibroelastosis led to the development of significant cardiac failure, with reversal of flow in the DV during atrial contraction. The observation of reversed flow in the DV could be explained by both end-stage heart failure and regurgitation due to valve insufficiency.

The association of an increased blood flow velocity in the fetal MCA with fetal anemia [17] has led to the accepted use of MCA Doppler instruments for the monitoring of pregnancies at the risk of fetal anemia, such as in cases of parvovirus infection [18]. The increase in Doppler blood flow velocity in anemic fetuses is thought to be related to decreased viscosity due to a decreased fetal red blood cell count and the increased cardiac output in the hyperdynamic circulation. However, further studies are required, since cases with severe anemia, which is usually present with the hydrops, can exhibit damage of the cardiac tissues that might hamper the reactional increase of cardiac output, and an increased fetal cardiac output in a fetus with parvovirus B19 infection could be masked by both severe myocarditis and cardiac dilatation. This might account for the poor prognosis of parvovirus B19 fetal hydrops in the second trimester of pregnancy, despite transfusional therapy attempts in the third trimester.

It is interesting that pericardial effusion, rather than ascites, was the predominant finding in the present case, because ascites is a much more common finding in cases of hydrops secondary to anemia. In cases of hydrops secondary to parvovirus B19, the anemia resulting from bone suppression leads to excessive hepatic erythropoiesis, which may result in portal hypertension and hypoproteinemia with consequent ascites. However, it is possible that myocarditis causing cardiac failure in the second trimester may cause massive pericardial effusion. One possibility is that in our case, the cardiac failure was secondary to myocarditis rather than anemia.

Today, there is still no consensus about the treatment for fetuses with hydrops following parvovirus B19 infection. The optimal antenatal treatment appears to be a combination of conservative management in selected cases [19, 20] and intervention such as intrauterine transfusion to correct the anemia [21, 22], plus thoracoamniotic shunting to treat pleural effusion or pericardial effusion in the more severe cases. Improvement of fetal hydrops and cardiac dysfunction has been reported after drug therapy with agents such as digitalis and in postnatal immunodeficient patients with chronic aplastic anemia, immunoglobulins have been successfully administered. In view of these therapeutic options, the termination of pregnancy is rarely indicated. It has recently been demonstrated that intrauterine transfusion is associated with an improved chance of survival in the presence of fetal hydrops or cardiovascular decompensation and it appears that intrauterine transfusion is indicated at an early stage of gestation in fetuses with more severe anemia [23–25]. However, fetuses with severe hydrops do not appear to tolerate intrauterine transfusions as well [26]. This treatment also remains controversial since anemia is not the only disorder involved in the pathogenesis. The intrauterine transfusion may be an ineffective therapeutic option for parvovirus B19 induced anemia, in the presence of a viral myocarditis. Indeed, myocarditis is sometimes responsible for the fetal demise and transfusion is not useful for such cases. Pregnant patients must be warned that such a treatment may correct the hydrops, but not the eventual sequelae of the associated myocarditis, which remains difficult to diagnose at such an early stage of gestation, and so the fetus may still have a poor outcome.

In conclusion, key parameters that should be monitored include pericardial effusion and MCA Doppler, as well as evidence of cardiomegaly, and DV blood flow. In addition, it appears that cardiac decompensation may be the main determining factor affecting fetal survival. It is hoped that the present case will increase awareness of the possibility of parvovirus as a cause of fetal hydrops due to myocarditis and that this may encourage studies to identify an effective management strategy.
